# Self-Microemulsifying Drug Delivery System: Formulation and Study Intestinal Permeability of Ibuprofen in Rats

**DOI:** 10.1155/2013/328769

**Published:** 2013-06-10

**Authors:** Bharat Bhushan Subudhi, Surjyanarayan Mandal

**Affiliations:** ^1^Department of Pharmaceutical Analysis, School of Pharmaceutical Sciences, SOA University, Khandagiri Square, \ Bhubaneswar, Orissa 751030, India; ^2^Mercury Pharmaceutical Limited, Gujarat, India

## Abstract

The study was aimed at developing a self-microemulsifying drug delivery system (SMEDDS) of Ibuprofen for investigating its intestinal transport behavior using the single-pass intestinal perfusion (SPIP) method in rat. *Methods*. Ibuprofen loaded SMEDDS (ISMEDDS) was developed and was characterized. The permeability behavior of Ibuprofen over three different concentrations (20, 30, and 40 µg/mL) was studied in each isolated region of rat intestine by SPIP method at a flow rate of 0.2 mL/min. The human intestinal permeability was predicted using the Lawrence compartment absorption and transit (CAT) model since effective permeability coefficients (*P*_eff_) values for rat are highly correlated with those of human, and comparative intestinal permeability of Ibuprofen was carried out with plain drug suspension (PDS) and marketed formulation (MF). *Results*. The developed ISMEDDS was stable, emulsified upon mild agitation with 44.4 nm ± 2.13 and 98.86% ± 1.21 as globule size and drug content, respectively. Higher *P*_eff_ in colon with no significant *P*_eff_ difference in jejunum, duodenum, and ileum was observed. The estimated human absorption of Ibuprofen for the SMEDDS was higher than that for PDS and MF (*P* < 0.01). *Conclusion*. Developed ISMEDDS would possibly be advantageous in terms of minimized side effect, increased bioavailability, and hence the patient compliance.

## 1. Introduction

In recent years, much attention has been focused on using emulsion drug delivery system for the purpose of improving the solubility and oral absorption of poorly water-soluble drugs [1–3]. SMEDDSs are defined as isotropic mixtures of natural or synthetic oils, solid or liquid surfactants, or, alternatively, one or more hydrophilic solvents and cosolvents/surfactants that have a unique ability of forming fine oil-in-water (o/w) microemulsions upon mild agitation followed by dilution in aqueous media, such as GI fluids [4]. SMEDDSs spread readily in the GI tract, and the digestive motility of the stomach and the intestine provide the agitation necessary for self-emulsification. SMEDDS is optically clear and thermodynamically stable system with a droplet size less than 100 nm. This system has been shown to improve absorption of drugs due to small droplet size and promotes intestinal lymphatic transport due to its specific components [5]. Ibuprofen (2-(4-(2-methylpropyl)phenyl) propanoic acid) is a nonsteroidal anti-inflammatory drug (NSAID) used for relief of symptoms of arthritis and fever. Ibuprofen is a poor water-soluble drug, and absorption in rats was shown to occur mainly from the intestine and to a lesser, though significant, extent from the stomach [6]. Since Intestinal permeability is necessary for oral administration, so in this study SMEDDS was developed to improve the solubility and oral absorption of Ibuprofen. Labrafil M 1944CS, Tween 80, and Transcutol P were used as oil phase, surfactant, and cosurfactant, respectively. The intestinal permeability of Ibuprofen was determined using the single-pass intestinal perfusion (SPIP) in rats since this method provides unique advantages of experimental control (e.g., compound concentration and intestinal perfusion rate), ability to study regional differences, an intact intestinal blood supply, and a functional intestinal barrier [7]. Intestinal permeability study of Ibuprofen through ISMEDDS using SPIP was a novel way to study the permeation behavior of Ibuprofen since it controls the experimental variables and also provides clear-cut idea of the permeation pattern in different regions of intestine.

## 2. Materials and Methods

### 2.1. Materials

Ibuprofen was obtained as gift sample from Abbott India Ltd., Goa, India. Capmul MCM, Labrafac CC, Cremophor EL, Cremophor RH 40, Labrafil M 1944CS, and Transcutol P were kindly provided by Gattefosse, France. Isopropyl Myristate (IPM), Tween 60, PEG 600, PEG 400, ethanol, and glycerol were purchased from National Chemicals (Baroda, India). Acetonitrile (HPLC grade), ammonium acetate, methanol (HPLC grade), and sodium citrate were purchased from Suvidhinath Laboratories (Baroda, India). All other chemicals were reagent grade.

### 2.2. Animals

Male albino rats (250 ± 20 g) were used for the comparative *in vivo *studies. The animals were maintained at temperature (25 ± 2°C) and humidity (60 ± 5%) and were supplied with food and water ad libitum. All experiments conducted on animals were approved by the Animal Ethical Committee and animal ethical committee registration no. 984/a/06/CPCSEA.

### 2.3. Solubility Studies

Solubility of Ibuprofen in various oils (Oleic acid, Isopropyl Myristate, Soya bean oil, Labrafil M 1944CS, and Capmul CMC), surfactants (Labrafac CC, Tween 60, Cremophor EL, and Cremophor RH 40), and cosurfactants (PEG 400, PEG 600, glycerol, and ethanol) was determined. A total of 5 mL of each of the selected vehicle was added to each cap vial with excess amount of Ibuprofen. After sealing, the mixture was heated at 40 ± 2°C in a water bath to facilitate the solubilization and then mixed using a vortex mixer. Mixtures were then shaken with orbital shaker at room temperature for 48 hrs. Then each vial was centrifuged at 4000 rpm for 5 min, and insoluble as well as soluble Ibuprofen was quantified by UV-VIS Spectrophotometer [8].

### 2.4. Pseudoternary Phase Diagram Study

**Figure 1:**
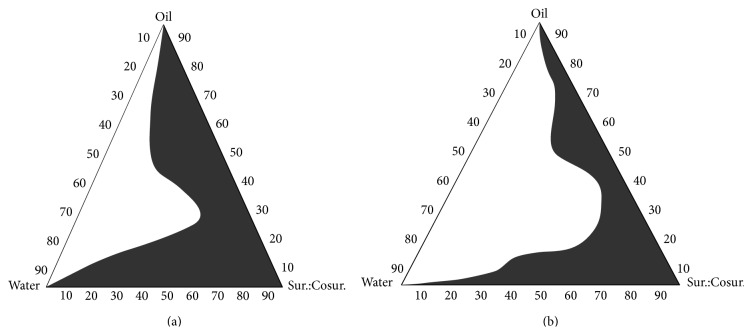
Shaded area of pseudoternary Phase diagram ((a) 3 : 1 and (b) 3.5 : 1 ratio of Sur: to Cosur.) representing the microemulsion area.

Pseudoternary phase diagrams were constructed to obtain the appropriate components and their concentration ranges that result in large existence area of microemulsion. For convenience, the phase diagrams were constructed by drawing “water dilution lines” representing increasing water content and decreasing surfactant-cosurfactant levels. If turbidity appeared followed by a phase separation, the samples were considered to be biphasic. If clear and transparent mixtures were visualized after stirring, the samples were considered monophasic. The samples were marked as points in the phase diagram as shown in Figure 1. The area covered by these points was considered to be the microemulsion existence region. After performing the solubility study and phase diagram study, components were selected for microemulsion formulation. The effect of Ibuprofen in the phase diagrams was also investigated [9].

### 2.5. Preparation of SMEDDS of Ibuprofen

ISMEDDS was prepared by emulsification method [10]. Ibuprofen was first dissolved in the premeasured volume of oil by stirring on a magnetic stirrer. A mixture of the surfactant and cosurfactant at a fixed ratio (v/v) was added to the above resulting mixture in order to develop SMEDDS. Emulsification time was also determined. The optimized ISMEDDS was also subjected for accelerated stability study.

### 2.6. Optical Clarity and Emulsification Time

Optical clarity of ISMEDDS was determined by the refractive index of the system and % transmittance. Refractive index was measured by an Abbe refractometer (Bausch and Lomb Optical Company, Rochester, NY, USA) by placing one drop of solution on the slide. Transparency of microemulsion formulation was determined by measuring percentage transmittance at 650 nm with purified water taken as blank through UV-VIS Spectrophotometer (UV-1601-220X, Shimadzu) [11]. 1 mL of the formulation was put into 100 mL of distilled water, and the dissolution time was measured as emulsification time.

### 2.7. Globule Size and Zeta Potential Measurement

Globule size and zeta potential of ISMEDDS were carried out by dynamic light scattering through Zetasizer 300 ZS-Nano, Malvern Instruments Corp., UK [12].

### 2.8. Single-Pass Intestinal Perfusion (SPIP) of the Rat Segments

Before the SPIP experiment, sufficient Krebs-Ringer's buffer solution was added to adjust the formulations to a designed concentration. The intestinal permeability experiments of ISMEDDS with different concentrations of Ibuprofen were investigated and compared to both PDS and MA of equivalent drug concentration. Preliminary studies are necessary before commencing the SPIP studies to ensure that the loss of drug from perfusion is due to absorption and not by other losses, that is, the binding of the intestinal wall or degradation [13]. Approximately 10 cm from the rat intestine was clipped; intestinal sacs were turned over and put into 50 mL Krebs-Ringer's buffer solution, in which Ibuprofen (20 *μ*g/mL) was incubated at 37 ± 2°C for 3 hrs. Chromatographic separation was achieved isocratically on a C18 column (Inertsil C18, 5 m, 150 mm × 4.6 mm) utilizing a mobile phase of acetonitrile/phosphate buffer/water (60 : 30 : 10, v/v, pH 7.0) at a flow rate of 1 mL/min with UV detection at 264 nm. Samples of 40 *μ*L were withdrawn at 3 hrs interval, diluted with mobile phase, and assayed by HPLC. SPIP studies were performed according to previously described methods [14–16]. After an overnight fast, male albino rats, weighing approximately 250 ± 20 gm, were anesthetized with 2 mg/kg i.m. injection of ketamine. Body temperature was maintained using a surgical lamp kept under the rat's cage. The abdomen was opened with a midline incision; an intestinal segment of approximately 10 cm was chosen and separated. Intestinal segment was then rinsed with isotonic saline (37°C) until the outlet solution was clear. Using an infusion pump the intestinal segments were perfused at a flow rate of 0.2 mL/min for 30 min with Krebs-Ringer's buffer solution. Then the pump was connected to the reservoir containing Ibuprofen loaded SMEDDS with the phenol red (marker). When the buffer solution was completely pushed out, time was set as *t* = 0. Each perfusion experiment lasted for 3 hrs, and the perfusate was quantitatively collected in 60, 90, 120, 135, 150, 165, and 180 min. Samples were stored in a refrigerator at −20°C until analysis by HPLC.

### 2.9. HPLC Analysis of Samples

All samples were diluted to 5 mL with the mobile phase and measured by HPLC (Shimadzu, Japan) system consisting of membrane degasser, binary solvent delivery system, a pump, a rheodyne injector equipped with a 20 *μ*L sample loop, and UV detector (Shimadzu, Japan) [17]. Aliquots of 20 *μ*L were injected onto the HPLC system which was used to analyze Ibuprofen at 264 nm. Chromatographic separations were achieved using an Hypersil ODS C18 column (250 × 4.6 i.d. × 5 *μ*m). The mobile phase used for the sample analysis was acetonitrile/phosphate buffer/water (60 : 30 : 10, v/v, pH 7.0) with the flow rate of 1.0 mL/min. Water absorption or secretion (flux) was measured by gravimetric method [18]. The net water flux (NWF) per cm of each segment was calculated using the following equation, and results were tabulated in Table 1:

(1) $$\begin{array}{c} \text{NWF}\left(\mu \text{L}/\min/\text{cm}\right)=\left[\left(1-\frac{{\text{CPR}}_{\text{out}}}{{\text{CPR}}_{\text{in}}}\right)\times \frac{Q_{\text{in}}}{L}\right]\times 1000, \end{array}$$

where CPR_in_ and CPR_out_ are inlet and outlet concentrations of phenol red, respectively, *Q*_in_ was the flow rate of the perfusion solution entering the intestinal segment, and *L* was the length of the intestinal segment (cm).

**Figure 2:**
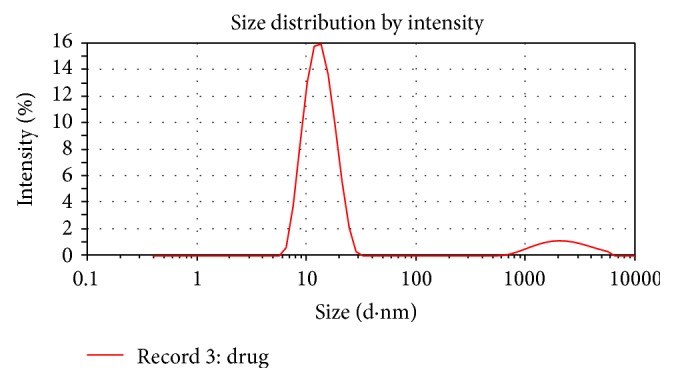
Globule size of Ibuprofen loaded SMEDDS indicating the nanosizing of the formulation.

NWF value will be negative, if intestinal lumen absorbs water, while NWF is positive if intestinal lumen secretes water; however, under normal condition, intestinal lumen absorbs water except when the high osmotic solution is administered [19]. The effective permeability (*P*_eff_) was calculated based on the inlet and outlet concentrations of Ibuprofen (*C*_in_ and *C*_out_, resp.), and result was shown in Figure 2. Steady state was considered to be achieved when the concentrations of phenol red and Ibuprofen were constant. The permeability values are calculated after steady state was achieved using the following equation [20]:

(2) $$\begin{array}{c} P_{\text{eff}}=\frac{\left[Q\ln{\left(C_{\text{in}}/C_{\text{out}}\right)}^{\prime }\right]}{2\pi rL}, \end{array}$$

where *Q* is the flow rate in mL/min, *r* is radius of the intestine (0.18 cm in rat and 1.75 cm in man), *L* is the length of the perfused intestinal segment (cm), and (*C*_in_/*C*_out_)′ is the concentration ratio corrected for fluid flux. Using ANOVA, the statistical significances of the differences among group means were assessed with the least significant difference (LSD) test, and a value of *P* < 0.05 was considered statistically significance.

## 3. Results

Labrafil M 1944CS was selected as the oil phase for formulation development because it provided higher solubility than other oils and oral compatibility. Tween 80 as surfactant and Transcutol P as cosurfactant were used on the basis of their drug solubilising capacity and might influence the tight junctions of the epithelial cells [21]. From the results of the pseudoternary phase diagram, 3 : 1 ratio of Tween 80 and Transcutol P was selected for microemulsion preparation since maximum microemulsion existing zone was observed as shown in Figure 1.

The optimal formulation of ISMEDDS was 4% Labrafil M 1944CS as oil, 21.5% Tween 80 as surfactant, and 7.5% Transcutol P as cosurfactant with globule size 44.4 nm ± 2.13 (PdI-0.266) as shown in Figure 2. PdI value was below 0.4 suggesting the monodisperse property of the formulation.

Zeta potential and drug content of the formulation were −9.19 mV ± 1.2 indicating the stability of the formulation and 98.86% ± 1.21, respectively. Developed SMEDDS was spontaneously forming microemulsion upon mild agitation in simulated gastric fluid at room temperature. % transmittance and refractive index of the resulted formulation were found to be 97.9% ± 1.31 and 1.337 ± 0.13 indicating the transparency as well as the nanosizing of the formulation. Moreover developed formulation was stable for 6 months at ambient temperature.

### 3.1. Validation of HPLC Assay

The regression equation for the concentration of Ibuprofen (ng/mL) v/s response in the perfusion fluid ranging from 50 ng/mL to 500 ng/mL was *P* = 91.32*C*–47.3 (*R* ^2^ = 0.9998). The mean recovery of Ibuprofen was 100.8 ± 1.13%. The intraday and interday RSDs were less than 2%.

### 3.2. Water Absorption or Secretion of Intestinal Lumens

The NWF for all intestinal segments was of negative value as listed in Table 1, which suggested that the water was mainly absorbed in the intestine tract. The NWF for the duodenum was significantly higher than that for the jejunum, ileum, and colon (*P* < 0.05), and NWF for the jejunum, ileum, and colon was not significantly varied (*P* > 0.05).

### 3.3. SPIP Studies

**Figure 3:**
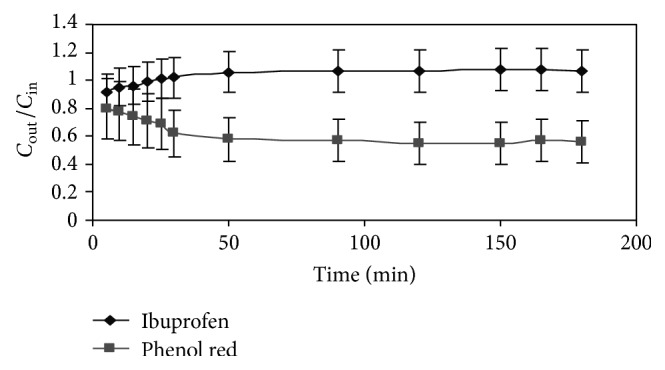
Concentration ratio of *C*_out_ and *C*_in_ with time for phenol red and Ibuprofen indicating the steady state permeability in intestine.

The amount of Ibuprofen was 99.32 ± 1.23% after incubated in intestinal sac with the Krebs-Ringer's buffer for 3 hrs, which was not significantly decreased which suggested that neither Ibuprofen markedly bound to the intestinal wall nor the potential metabolites were formed. The intestinal permeability of Ibuprofen was studied as a function of concentration in each segment of the intestine, and 30 *μ*g/mL concentration was chosen because of its limited aqueous solubility. Steady state is confirmed by plotting the concentration of Ibuprofen and phenol red versus time as shown in Figure 3.

Steady state, which was assessed by the constant concentrations of both phenol red and Ibuprofen, was reached about 50 min after the beginning of the perfusion. The permeability values were calculated only after steady state was achieved in the experiments. The permeability values of SMEDDS for each intestinal segment and concentrations are listed in Table 2. *P*_eff_ in the jejunum at 10 *μ*g/mL (0.388 ± 0.021 cm/s) was significantly higher than *P*_eff_ at 20 *μ*g/mL (0.229 ± 0.037 cm/s, *P* < 0.01). There was no statistical difference in *P*_eff_ at three investigated concentrations in other segments (including duodenum, ileum, and colon). Comparisons across each segment revealed that *P*_eff_ at each concentration in colon was higher than all other segments (*P* < 0.01). Moreover, the permeability values in duodenum, jejunum, and ileum were not statistically different (*P* > 0.05).

**Figure 4:**
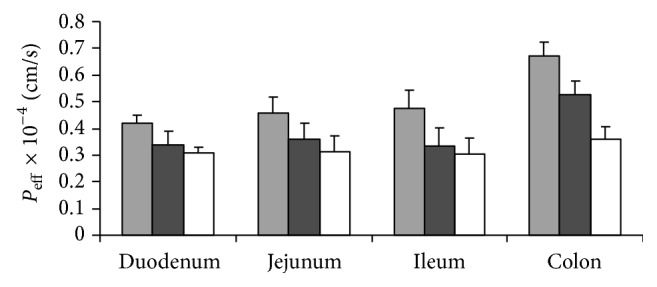
Comparison of *P*_eff_ of the SMEDDS, PDS, and MF in different intestinal segments (*n* = 4) at concentration of the Ibuprofen as 10 *μ*g/mL.

Permeability values of Ibuprofen in ISMEDDS, PDS, and MF for each segment at 10 *μ*g/mL were shown in Figure 4. *P*_eff_ of ISMEDDS was significantly higher than PDS and MF in each segment (*P* < 0.01). Except for the duodenum (*P* < 0.05), *P*_eff_ of microemulsion drug delivery system and MF in the jejunum, ileum, and colon did not all reach statistical difference (*P* > 0.05).

## 4. Discussion

Characterization data and microemulsifying study in simulated gastric fluid revealed that the developed ISMEDDSs was of nanosize, stable and was forming microemulsion spontaneously. *P*_eff_ in the colon at each concentration was significantly higher than those in other segments of rat intestine as shown in Table 2. There are various reports showing the regional difference in the expression and activity of P-glycoprotein throughout the gastric intestinal tract. The order of the expression and activity of P-glycoprotein is ileum > duodenum and jejunum > proximal and distal colon. As a multidrug resistance-reversing agent, the intestinal absorption of Ibuprofen was limited due to the presence of the P-glycoprotein throughout the intestinal tract [22]. The lower expression activity of P-glycoprotein was conduced to be increasing the permeability of Ibuprofen in colon as compared to other sections (Table 2). It has been shown that there is a selectivity of permeation depending of the gut sections. Moreover, small intestine has more cationic selectivity, whereas the colon has more anionic selectivity [23]. Another possible mechanism for higher *P*_eff_ values in colon was due to the higher crypt surface area in the colonic mucosa compared with the small intestine which could account for its high paracellular permeability. The extent of drug absorption in human can be predicted from rat intestinal permeability experiments. Human intestinal permeability values may be estimated using the Lawrence compartmental absorption and transit (CAT) model [24]:

(3) $$\begin{array}{c} \text{Fa}\left(\text{fraction}\;\text{of}\;\text{dose}\;\text{absorbed}\right)=1-{\left[1+0.54\;P_{\text{eff}}\left(\text{man}\right)\right]}^{-7},\\ P_{\text{eff}}\left(\text{man}\right)=3.6\times P_{\text{eff}}\left(\text{rat}\right)+0.03\times 10^{-4}. \end{array}$$

The estimated fractions of Ibuprofen absorbed for ISMEDDS, MF, and PDS were 85.5 ± 1.9%, 59.3 ± 3.1%, and 81.2 ± 2.2%, respectively. It suggested that the estimated oral absorption in human for microemulsion drug delivery systems would be higher than that for PDS and MF (*P* < 0.01). SMEED is known to improve the oral absorption of lipophilic drugs. The main mechanism reported includes increasing membrane fluidity, opening tight junction, inhibiting P-glycoprotein and/or CYP450 by surfactants, and stimulating lipoprotein/chylomicron production by lipid [25].

## 5. Conclusions

Self-microemulsifying drug delivery system of Ibuprofen was a monodisperse stable system capable of forming microemulsion systems in simulated gastric fluid upon mild agitation which will provide sustained drug release in intestinal part. % assay was above 95% indicating the system's suitability with drug. Ibuprofen loaded SMEDDS and its dilutions were stable. The *P*_eff_ in jejunum at 10 *μ*g/mL was significantly higher than that at 20 *μ*g/mL (*P* < 0.01). Compared to the jejunum, duodenum, and ileum, the higher *P*_eff_ in colon was observed at the same concentration. The estimated absorption of Ibuprofen in human for the microemulsion drug delivery system was higher than that for PDS and MF (*P* < 0.01).

## Figures and Tables

**Table 1:** The net water flux (NWF) in each intestinal segment (*n* = 4).

Time (min.)	Duodenum	Jejunum	Ileum	Colon
60	−0.584 ± 0.121	−0.144 ± 0.175	−0.102 ± 0.056	−0.129 ± 0.018
80	−0.555 ± 0.212	−0.117 ± 0.128	−0.238 ± 0.078	−0.197 ± 0.056
100	−0.789 ± 0.374	−0.209 ± 0.124	−0.237 ± 0.032	−0.426 ± 0.244
130	−0.672 ± 0.221	−0.206 ± 0.226	−0.345 ± 0.057	−0.298 ± 0.057

**Table 2:** Comparison of effective permeation coefficients (*P*_eff_ × 10^−4^) of different intestinal segments with three concentrations. Results are given as mean ± SD (*n* = 4).

Conc. of Ibuprofen (*μ*g/mL)	*P*_eff_ in duodenum (cm/s)	*P*_eff_ in jejunum (cm/s)	*P*_eff_ in ileum (cm/s)	*P*_eff_ in colon (cm/s)
10	0.421 ± 0.032	0.458 ± 0.044	0.475 ± 0.052	0.671 ± 0.067
20	0.388 ± 0.035	0.434 ± 0.046	0.439 ± 0.067	0.598 ± 0.062
30	0.409 ± 0.064	0.389 ± 0.043	0.441 ± 0.087	0.498 ± 0.044

## References

[1] Shah N. H., Carvajal M. T., Patel C. I., Infeld M. H., Malick A. W. (1994). Self-emulsifying drug delivery systems (SEDDS) with polyglycolyzed glycerides for improving in vitro dissolution and oral absorption of lipophilic drugs. *International Journal of Pharmaceutics*.

[2] Wu W., Wang Y., Que L. (2006). Enhanced bioavailability of silymarin by self-microemulsifying drug delivery system. *European Journal of Pharmaceutics and Biopharmaceutics*.

[3] Kommuru T. R., Gurley B., Khan M. A., Reddy I. K. (2001). Self-emulsifying drug delivery systems (SEDDS) of coenzyme Q10: formulation development and bioavailability assessment. *International Journal of Pharmaceutics*.

[4] Tang J.-L., Sun J., He Z.-G. (2007). Self-emulsifying drug delivery systems: strategy for improving oral delivery of poorly soluble drugs. *Current Drug Therapy*.

[5] Charman W. N., Stella V. J. (1991). Transport of lipophilic molecules by the intestinal lymphatic system. *Advanced Drug Delivery Reviews*.

[6] Kapsi S. G., Ayres J. W. (1999). Processing factors in development of solid solution formation of Ibuprofen for enhancement of drug dissolution and bioavailability. *International Journal of Pharmaceutics*.

[7] Salphati L., Childers K., Pan L., Tsutsui K., Takahashi L. (2001). Evaluation of a single-pass intestinal-perfusion method in rat for the prediction of absorption in man. *Journal of Pharmacy and Pharmacology*.

[8] Kaewnopparat N., Kaewnopparat S., Jangwang A., Maneenaun D., Chuchome T. (2009). Increased solubility, dissolution and physicochemical studies of curcumin-polyvinylpyrrolidone K-30 solid dispersions. *World Academy of Science, Engineering and Technology*.

[9] Aboofazeli R., Lawrence M. J. (1994). Investigations into the formation and characterization of phospholipid microemulsions. II. Pseudo-ternary phase diagrams of systems containing water-lecithin-isopropyl myristate and alcohol: influence of purity of lecithin. *International Journal of Pharmaceutics*.

[10] Patel B. M., Mandal S., Rajesh K. S. (2012). Formulation and kinetic modeling of curcumin loaded intranasal mucoadhesive microemulsion. *Journal of Pharmacy and Bioallied Sciences*.

[11] Date A. A., Nagarsenker M. S. (2008). Design and evaluation of microemulsions for improved parenteral delivery of propofol. *AAPS PharmSciTech*.

[12] Patel D., Sawant K. K. (2007). Oral bioavailability enhancement of acyclovir by self-microemulsifying drug delivery systems (SMEDDS). *Drug Development and Industrial Pharmacy*.

[13] Cook T. J., Shenoy S. S. (2003). Intestinal permeability of chlorpyrifos using the single-pass intestinal perfusion method in the rat. *Toxicology*.

[14] Zakeri-Milani P., Valizadeh H., Tajerzadeh H. (2007). Predicting human intestinal permeability using single-pass intestinal perfusion to rat. *Journal of Pharmacy and Pharmaceutical Sciences*.

[15] You J., Li Q.-P., Yu Y.-W., Cui F.-D. (2004). Absorption of zedoary oil in rat intestine using in situ single pass perfusion model. *Acta Pharmacologica Sinica*.

[16] Grassi M., Cadelli G. (2001). Theoretical considerations on the in vivo intestinal permeability determination by means of the single pass and recirculating techniques. *International Journal of Pharmaceutics*.

[17] Tsao J. C., Savage T. S. (1985). High-performance liquid chromatographic determination of ibuprofen in bulk drug and tablets. *Drug Development and Industrial Pharmacy*.

[18] Sutton S. C., Rinaldi M. T., Vukovinsky K. E. (2001). Comparison of the gravimetric, phenol red, and 14C-PEG-3350 methods to determine water absorption in the rat single-pass intestinal perfusion model. *AAPS PharmSci*.

[19] DeSesso J. M., Jacobson C. F. (2001). Anatomical and physiological parameters affecting gastrointestinal absorption in humans and rats. *Food and Chemical Toxicology*.

[20] Fagerholm U., Johansson M., Lennernäs H. (1996). Comparison between permeability coefficients in rat and human jejunum. *Pharmaceutical Research*.

[21] Mandal S., Mandal S. S., Rajesh K. S. (2011). Development, characterization and evaluation of microemulsion gel for transdermal delivery of furosemide. *Journal of Pharmacy Research*.

[22] Ikegawa T., Ushigome F., Koyabu N. (2000). Inhibition of P-glycoprotein by orange juice components, polymethoxyflavones in adriamycin-resistant human myelogenous leukemia (K562/ADM) cells. *Cancer Letters*.

[23] Ma T. Y., Hollander D., Erickson R. A., Truong H., Nguyen H., Krugliak P. (1995). Mechanism of colonic permeation of inulin: Is rat colon more permeable than small intestine?. *Gastroenterology*.

[24] Yu L. X., Amidon G. L. (1999). A compartmental absorption and transit model for estimating oral drug absorption. *International Journal of Pharmaceutics*.

[25] Sha X., Yan G., Wu Y., Li J., Fang X. (2005). Effect of self-microemulsifying drug delivery systems containing Labrasol on tight junctions in Caco-2 cells. *European Journal of Pharmaceutical Sciences*.

